# Improving the quality of teacher education for sustainable development of Taiwan's education system: A systematic review on the research issues of teacher education after the implementation of 12-year national basic education

**DOI:** 10.3389/fpsyg.2022.921839

**Published:** 2022-09-09

**Authors:** Ru-Jer Wang, Yi-Haung Shih

**Affiliations:** ^1^Department of Education, National Taichung University of Education, Taichung, Taiwan; ^2^Department of Early Childhood Education and Care, Minghsin University of Science and Technology, Hsinchu, Taiwan

**Keywords:** 12-year basic education, preservice teacher education, systematic review, preservice teachers, teacher education

## Abstract

In this study, the researchers analyzed the issues in teacher education research after the implementation of the 12-Year Basic Education policy and developed suggestions to improve the quality of teacher education in Taiwan. The researchers used Taiwan's *Database of Journal Citations in Science Education* to collect studies on teacher education published from 2015 to 2019 and analyzed the issues described therein. Fifteen categories of issues described therein. Regarding our suggestions for future research on teacher education, this study recommends strengthening the body of literature on the following themes: (1) the professional development of preservice teachers, (2) teacher education for special education, (3) quality assurance for teacher education, (4) the career development of preservice teachers, (5) teacher education for experimental education and rural schools, (6) the sociology of teacher education, and (7) the operation of teacher education centers. Our findings may serve as a reference in efforts to improve the quality of teacher education, and promoting the sustainable development of Taiwan's education system.

**Systematic review registration:**

http://w1.dorise.info/JCSE/.

## Introduction

### How teacher education research can improve the quality of teacher education

The implementation of education affects a country's development and success, and a key component of an effective education system is the cultivation of high-quality teachers. The quality of education often depends on the quality of teachers, and teachers' professional development is closely linked to teaching effectiveness and student learning outcomes. In discussions on teacher education reform, high-quality teachers are often considered the foundation of student achievement, and the cultivation of high-quality teachers depends on research-based teacher education. Practice and theory must be integrated to ensure high-quality teacher education. Theory can improve practice, and practice can be used to revise theory; that is, teacher education theory can guide the implementation and thus improve the quality of teacher education. The development of the theoretical basis of high-quality teacher education requires extensive research on teacher education. Studies on teacher education can contribute to the development of the theoretical basis of teacher education (e.g., preservice programs), promote best teaching practices in the workplace, and enhance the quality of teacher education (Shor and Freire, 1987; Darling-Hammond, 2006; Allen, 2008; Waks, 2008; Shih et al., 2020).

### Teacher education after the implementation of 12-year national basic education in Taiwan

Taiwan's 12-Year Basic Education was first implemented in August 2014, and the Ministry of Education announced the *Curriculum Guidelines for 12-Year Basic Education: General Guidelines* (hereinafter referred to as the New Curriculum) in November 2014. The New Curriculum reflects the idea that the 12-year basic education curriculum guidelines should be based on the principle of holistic education, incorporating the ideas of “taking initiative”, “engaging in interaction”, and “seeking the common good”. The practice of the New Curriculum is based on “core competency” as its main axis and consists of three dimensions: “autonomous action”, “communication and interaction”, and “social participation” (Ministry of Education, 2014). In August 2019, the New Curriculum was formally implemented in Taiwan's education system. During the 5 years between the initial implementation of 12-year basic education and the formal implementation of the New Curriculum, many major reforms were adopted in the system.

In response to the development of the competency-based New Curriculum, some reforms were made to Taiwan's preservice teacher education courses in 2018. The Ministry of Education published the *Guide to the Professional Competence of Teachers in the Republic of China: Preservice Teacher Education Stage and Preservice Teacher Education Course Benchmarks*, with the aim of establishing the contents of competency-based preservice education courses and granting autonomy to teacher education universities for preservice education courses to ensure that preservice teachers attain professional knowledge and attitudes and that the courses emphasize educational best practices to develop the enthusiasm and sense of responsibility of preservice teachers. In addition, the guide promotes theoretical and practical methods for evaluating the professional teaching abilities of preservice teachers in preservice teacher education courses. Finally, the guide stipulates that teacher education universities must encourage preservice teachers to complete teacher qualification examinations and must conduct teacher education evaluations to assess the teachers' achievement and ensure the quality of teacher education courses (Ministry of Education, 2018a).

In addition to reforming preservice teacher education courses, Taiwan's Ministry of Education also reformed the teacher qualification examination in 2017. Preservice teachers must pass the teacher qualification examination before participating in practical education training to demonstrate their professional ccompetence, which constitutes the evaluation framework and content of the examination. In 2018, the Ministry of Education implemented a new practical education training policy that emphasizes the evaluation of preservice teachers' files and the process of evaluation, requiring cooperating teachers and instructors to use standards-based evaluation to assess the performance of student teachers and encouraging student teachers to travel abroad for practical education training.

Other policies designed to support the 12-Year Basic Education system include subsidizing upgrades of teacher education programs at teacher education universities, improving the teaching skills of government-sponsored preservice teachers in remote areas, increasing the indigenous language expertise of government-sponsored indigenous preservice teachers, cultivating preservice teachers with expertise in national languages and experimental education, providing professional training for special education teachers to increase their subject expertise, and promoting bilingual teacher education (Ministry of Education, 2018b,c, 2019a,b).

After the implementation of 12-Year Basic Education in Taiwan, various reforms to the teacher education system were rapidly enacted. These reforms included the implementation of competency-based preservice teacher education courses, adjustment of the teacher qualification examination and practical education system to improve the evaluation of preservice teachers, and other supporting measures related to 12-Year Basic Education. The enactment of these reforms has raised questions regarding the state of research on teacher education, including what themes such research has focused on to date, whether the studies conducted to date can effectively guide the development of Taiwanese teacher education, and which other areas of teacher education–related research require further development.

In response to the aforementioned questions, this study explored the issues discussed in teacher education research after the implementation of 12-Year National Basic Education in Taiwan and developed suggestions to further improve the body of literature. The researchers used Taiwan's *Database of Journal Citations in Science Education* to collect research related to teacher education from 2015 to 2019 and therein summarize and analyze the issues covered in the studies. Second, some recommendations for future research on teacher education in Taiwan are provided. The findings of this study may serve as a reference in efforts to improve teacher education and Taiwan's education system.

## Issues in research on teacher education in Taiwan

The researchers used the documentary analysis method, which involves exploring various arguments and events by applying deductive and inductive logic. We analyzed studies related to teacher education after the implementation of 12-Year National Basic Education, and used deductive and inductive logic to explore the issues discussed therein (Wang, 1991; Shih, 2020).

This study collected a total of 92 studies related to teacher education from 2015 to 2019 from Taiwan's *Database of Journal Citation in Science Education*. These studies were related to the *Teacher Education Act, Teacher Education White Paper of the Republic of China, Teacher Professional Standards, Guide to the Professional Competence of Teachers in the Republic of China: Preservice Teacher Education Stage and Preservice Teacher Education Course Benchmarks*, and other regulations and documents. The 92 studies were divided into 15 issues and examined to assess the status of research on teacher education after the implementation of 12-Year Basic Education in Taiwan.

**Figure 1:**
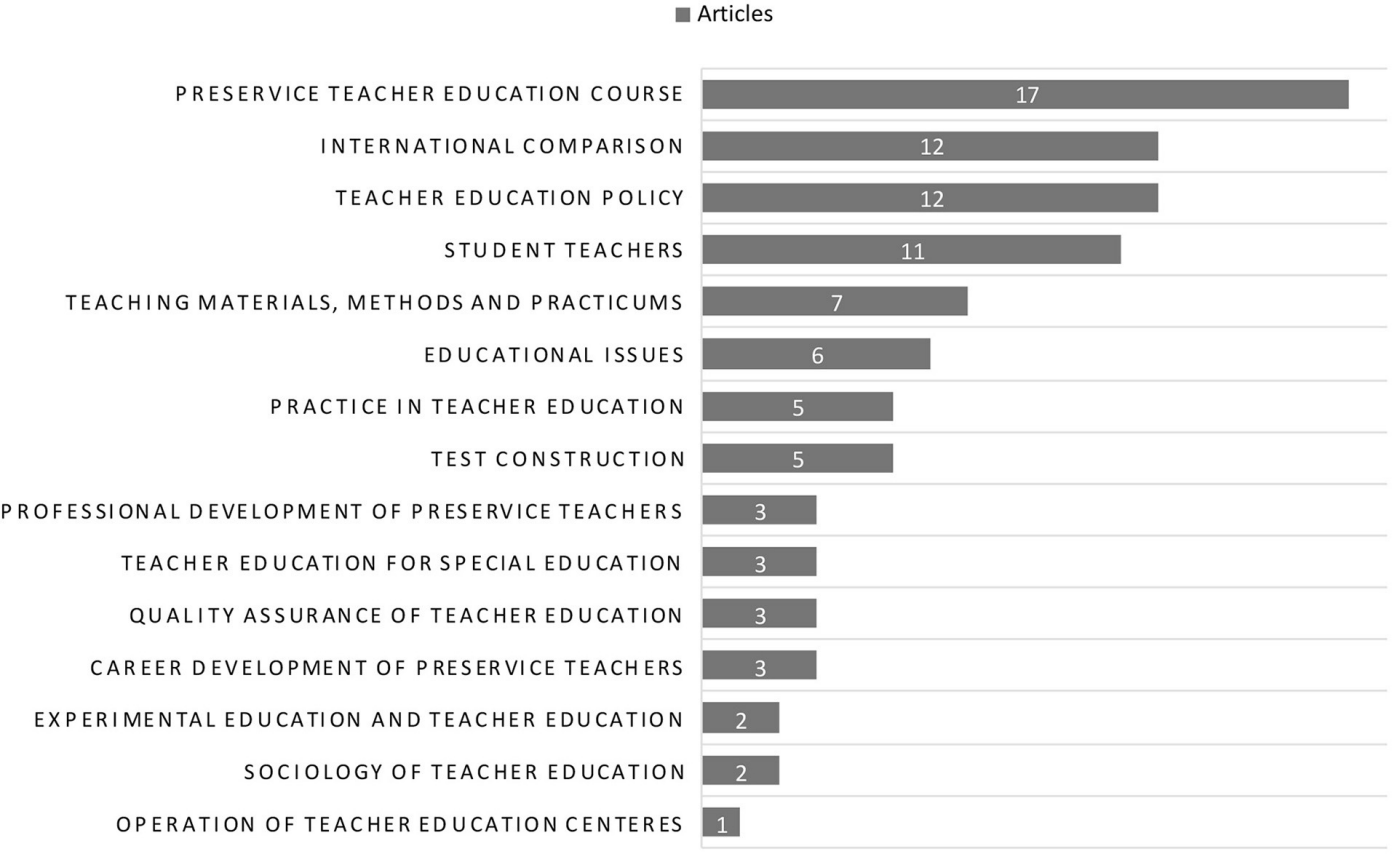
Research issues and numbers of articles related to teacher education in Taiwan from 2015 to 2019. The researchers.

The 15 issues are as follows: (1) preservice teacher education courses; (2) international comparison; (3) teacher education policy; (4) student teachers; (5) teaching materials, methods, and practicums; (6) educational issues; (7) practices in teacher education; (8) test construction; (9) professional development of preservice teachers; (10) teacher education for special education; (11) quality assurance of teacher education; (12) career development of preservice teachers; (13) experimental education and teacher education; (14) sociology of teacher education; and (15) operation of teacher education centers. The number of articles related to each issue is presented in Figure 1.

### Preservice teacher education courses

Regarding the studies on teacher education courses, one study discussed the development strategy of the teacher education system through the lens of the religious concept of servant leadership (Chang, 2015). Another study explored how to plan preservice teacher education courses for secondary technical and vocational education from the perspective of standards-based teacher education (Hsu, 2015).

One study examined the possibility of offering capstone courses, which are widely offered by universities, as part of the teacher education system (Kuo and Chen, 2017). Another work discussed the “Educational Issue” curriculum design model in preservice teacher education courses (Chang and Lin, 2016). Some studies have focused on the development of university courses that were transformed into elementary school preservice teacher education courses and the development of teaching aids (Lu and Li, 2016; Lu, 2017). Another study explored the design and practice of remedial English teaching courses to construct the professional identity of preservice English teachers (Chien, 2016). Another work aimed to improve the effectiveness of reading comprehension and teaching skills of preservice teachers (Chang, 2017).

Another study required preservice teachers to construct a problem-based practical community to enhance the learning effectiveness of preservice (Wang, 2016). The research on teacher education for 12-Year Basic Education has focused on competency-based curriculum reform, including discussing how teacher education universities can plan preservice teacher education courses that meet the requirements of the New Curriculum (Fwu, 2018). One study discussed how to design and implement the curricula of competency-based courses to ensure teachers' comfort with differentiated instruction and diversified learning content (Chen, 2018).

In response to the New Curriculum of 12-Year Basic Education, one study used the prediction–observation–explanation teaching method to design experimental courses and evaluate whether experimental courses can improve students' scientific explanation abilities (Lu and Ku, 2019). Another study used a combination of an online teaching cases observation course and face-to-face workshops in case teaching to encourage preservice teachers' participation in learning (Hung et al., 2018).

Regarding the studies of early childhood education, one study used example stories to improve preservice teachers' educational beliefs (Chen T. T., 2016; Chen Y.-H, 2016). Another study incorporated service learning into the “Early Childhood Visual Art Education” course at a department of early childhood educare, which encouraged the students to integrate theory and practice (Chen, 2015). Yet another study employed cognitive apprenticeship and peer assessment learning modules and invited senior preschool teachers to guide the learning of preservice teachers (Sun, 2015). Chen (2017) incorporated interdisciplinary integration and problem-based learning in the service learning of students in a department of early childhood education and care Chen (2017).

### International comparison

Studies exploring teacher education systems in different countries can serve as references for reflecting on Taiwan's teacher education system. Lu (2018) discussed the organization of preservice teacher internship courses in the United States and explored how it has informed Taiwan's teacher education system. Lin (2017) discussed teacher training systems for non-state operating models. Another work compared the teacher education systems for special education in Taiwan and Japan (Hsiao, 2017). Gao and Wang (2015) analyzed Thailand's teacher education system and teacher quality management mechanism. Another study used the European Jena teacher education system as an example to explain the teacher development policy of the United Nations and explored how to improve students' learning (Chen and Hsu, 2017).

Yu (2018) discussed the characteristics of the National Core Curriculum for Basic Education in Finland and the effect of the curriculum on teacher education, and a few other studies have explored the characteristics of Finnish teacher education (Liu, 2015; Wang and Hsu, 2015). A more macroscopic study explored how to reform physical teacher education courses on the basis of different historical and cultural backgrounds from a global perspective (Wei and Shy, 2016). (Chen T. T., 2016; Chen Y.-H, 2016) focused on the international development trend of moral education and explored how to cultivate teaching ethics among preservice teachers.

### Teacher education policy

Taiwan's implementation of 12-Year Basic Education. It has changed very rapidly and transformed teacher education nationwide, and researchers have conducted some studies on related policies. Hsieh (2016) reviewed the development of the Taiwanese teacher education system from 1994 to 2014 and discussed the changes in the system enacted during different periods and the associated problems faced by teacher education institutions. Another study reported that standards-based teacher education can be used to ensure the organization and innovation of procedures in teacher education from a legal perspective (Wang, 2016).

After the implementation of the *Teacher Education Act* in 1994, education of secondary school teachers was transferred from normal universities to general universities in Taiwan. Since then, some changes have been made in the selection system for preservice teachers, preservice teacher education courses, teacher qualification examinations, and practical education training (Yen, 2019). Some researchers argue that teachers must systematically acquire professional knowledge to become “responsible qualified teachers”, that the competence of student teachers must be improved when they enter the education workforce, and that teachers must be required to attain high scores on teacher qualification examinations (Cheng, 2015).

One study discussed the key factors affecting preservice teachers' ability to obtain teaching certificates and advocated that preservice teachers' practical teaching experience should be strengthened systematically (Huang et al., 2017). Another study discussed the effectiveness of the Ministry of Education's scholarship program for excellent teacher education (Wen et al., 2016). Another study explored the unique government-funded system of teacher education in Taiwan, its continuation after the diversification of teacher education, and its functions of attracting excellent preservice teachers and providing excellent teachers for rural schools (Li et al., 2016; Huang, 2019).

One study considered the roles of a teacher education system from the perspective of youth's global mobility (Yen, 2016). This view is reflected in the foreign educational internship, practical education training, and international Schweitzer program promoted by the Ministry of Education after 2016.

Lin (2019) examined the *Teacher Education White Paper of the Republic of China* published in 2012, the *Guides for Professional Standards of Teachers* published in 2016, 12-Year Basic Education, and the 2018 *Guide to the Professional Competence of Teachers in the Republic of China: Preservice Teacher Education Stage and Preservice Teacher Education Course Benchmarks* and reflected on the failure of these measures to keep pace with the times. The aforementioned problems are affecting current research on teacher education and do not effectively link policy development and dialogue between practitioners (Wu, 2018).

The *Guide to the Professional Competence of Teachers*, which was published by the Ministry of Education in 2018, has strongly affected the structure of competency-based specialized curricula. One study constructed benchmarks for specialized curricula for preservice secondary school art teachers (Cheng et al., 2017).

Chen and Chang (2017) explored the process of adopting Teaching Games for Understanding for student teachers of physical education in middle schools. Only one study has examined the problems facing student teachers in early childhood education and potential practical solutions to these problems (Chang et al., 2017).

### Student teachers

Some research on student teaching focuses on professional competence. Huang and Wu (2015) explored the acquisition, use, and importance of professional knowledge and ability for student teachers. Another study focused on practical education training and the theme of administrative ethics among student teachers that is relevant to various types of school administrative work (Chen, 2015). Another study used self-regulation learning to cultivate student teachers' work value and self-learning ability and developed student teachers' learning strategies for self-regulation (Lin et al., 2018).

Regarding research on the career development of student teachers, only one study reported that teachers' beliefs and achievement goals have a positive and direct effect on creative teaching beliefs but willingness to teach has a negative effect on creative teaching beliefs (Hsiao, 2018).

In recent years, three studies have investigated cooperating teachers who guide student teachers at their educational sites. One study explored the progress of student teachers in teaching ability and class management during practical education training. During this stage, cooperating teachers have the greatest influence on the professional development of student teachers (Lin et al., 2015). Another study explored the experiences and professional development of cooperating teachers when they guide student teachers (Yu, 2015). Huang and Wei (2016) promoted the quality of practical education training and proposed the establishment of a cooperating teacher certification system.

In 2017, the Ministry of Education began to promote foreign practical education training, foreign education internships, and the international Schweitzer program, which were intended to cultivate teachers' international outlooks, language skills, and willingness to work in overseas schools (Ministry of Education, and National Taiwan Normal University, 2019). Since 2016, only one study has used overseas practical education training experience to examine the importance of education and cultural systems in different countries (Lyu, 2016).

### Teaching materials, methods and practicums

Studies on teaching materials, methods and practicums have mainly explored the application of different teaching methods in various subjects to enhance the teaching ability and attitudes of preservice teachers. For example, Pan (2018) explored the motivation, expectations, and learning experience of preservice elementary school teachers studying “Integrated Activities Teaching Materials and Methods”, and Chen (2018) emphasized multiliteracy in “English Teaching Materials and Methods”. Another study applied activity theory to mathematics teaching and discussed the conditions of preservice teachers in the process of application (Chen T. T., 2016; Chen Y.-H, 2016). Su (2016) planned a “mathematics practicum” to enhance the ability of preservice teachers to express, discuss, and review, and another study used problem-based learning to develop preservice teachers' critical thinking and problem-solving skills (Chang and Lin, 2016). Finally, Huang (2017) developed enhanced strategies for teaching physical education creatively, employing different teaching methods, and increasing the successful teaching experiences of preservice teachers.

### Educational issues

A language curriculum for new resident preservice teachers was proposed in Taiwan's New Curriculum. The need for high-quality new resident preservice teachers is an urgent problem that must be addressed (Hwang and Wu, 2019). In addition to the education of new resident language teachers, some studies have also focused on how to design and implement multicultural preservice education courses (Ho and Hsieh, 2019). The education of Taiwan's new resident language teachers depends on whether preservice teacher education courses can provide suitable curricula and textbooks, and a comprehensive policy package has been proposed to enable teacher education universities to steadily educate new resident language teachers (Chang, 2017).

In addition, one study suggested that teacher education should focus on gender equality education–related courses and actively construct the core concepts of gender education, curriculum objectives, and the development of teaching materials and methods (Yang, 2015). Another study explored how to cultivate the life education literacy of preservice teachers in preservice teacher education courses and incorporate life education themes into teaching activities and course content for preservice teachers (Li and Lo, 2015).

### Practice in teacher education

In recent years, Taiwan's teacher education practices have been subject to increased attention. One study explored the manufacturing and teaching of English picture books (Tsai, 2015); another study investigated the implementation of teaching plan design, trial teaching, and reflection experience in environmental education (Wang, 2020). Yet another study focused on how professional teachers can develop their own knowledge of reflection practice when they guide student teachers (Chen et al., 2019). Using a self-study method, Chiou (2016) discussed how teacher trainers and preservice teachers learn together to construct a classroom learning experience.

From 2015 to 2019, the Ministry of Education required preservice teachers to complete at least 54 h of field-based experience before graduation. The purpose was to strengthen the connection between preservice teachers' professional education and school education to achieve the integration of knowledge and action. One study proposed creating alternative methods for active trials to improve the quality of field-based experience (Fwu and Hwang, 2016).

### Test construction

The goal of the *Teacher Education White Paper of the Republic of China* is to strengthen preservice teachers' selection and improve preservice teacher education courses. Thus, after 2014, the Ministry of Education cooperated with National Taiwan Normal University to develop the Computerized Teacher Aptitude Assessment (TAA), which comprises the “Work Values Scale for Teachers”, “Situational Judgment Tests for Teachers”, and “Personality Scale for Teachers”. The TAA involves conducting tests close to the actual teaching site; exploring students' teaching quality, emotions, and behavioral performance in the context of work; and supplementing the content that cannot be measured using cognitive test performance and academic achievement. The TAA can be used for preservice teacher selection and to evaluate whether preservice teacher education courses are useful. Finally, the TAA can also be used to evaluate the performance of student teachers (Ministry of Education, and National Taiwan Normal University, 2019).

The development of tests related to teacher education has emphasized the use of multiple assessments and of indicators other than academic achievement (Chao et al., 2018). One study focused on preservice teachers' motivation for taking preservice teacher education courses (Chang, 2017). Another study assessed the professional performance of preservice teachers (Ma and Lin, 2015). Other studies have focused on constructing assessments or scales for preservice teachers' teaching performance (Chiu and Chan, 2015; Huang and Chang, 2018).

### Professional development of preservice teachers

Regarding the professional development of preservice teachers, Hwang (2015) focused on the construction of teacher images and the ideals of teacher education. Tang and Zeng (2017) observed that in a standards-based teacher education system, preservice education courses still focus on the traditional professional abilities of teachers, and few courses train teachers to cooperate with others and cultivate their own research capabilities. The study also noted that the elective system could affect standards-based teacher education. From the perspective of teacher educators, Sung (2017) contended that the development of professional ethics for preservice teachers should be based on understanding the context and nature of education reform and clarifying the goals and ideals of the teaching profession.

### Teacher education for special education

In response to the Ministry of Education's 2016 blueprint for standards-based cultivation of teachers, one study surveyed preservice teachers in special education to evaluate their professional performance in the categories of professional ethics, collaborative deliberation, curriculum and instruction, classroom teaching, and educational professionalism (Wu et al., 2019).

Taiwan's preservice education programs have integrated introductory courses on special education to enhance the knowledge of preservice teachers. One study used case-based instruction in a course to enhance preservice teachers' learning attitudes and effectiveness (Lee and Huang, 2016). Another study attempted to use embedded teaching through the naturalistic teaching approach to enhance preservice teachers' ability to support special education students in the context of integrated education (Tseng and Yang, 2018).

### Career development of preservice teachers

Preservice teachers' career development is related to the achievements of licenses and the competency development, and whether preservice teachers engage in education after graduation is also a “key indicator” for teacher education evaluation in Taiwan. One study interviewed with mature preservice teachers in their 30s to explore their characteristics of psycho-sociologically, and analyze their needs, and provide a more flexible and diversified development for teacher education system (Chiang, 2015). One study explored the “Project for Excellent Teacher Education Scholarship” to examine the teaching preparedness of student teachers and their teaching performance (Huang, 2016a). One study explored the motivation and needs of preservice teachers' career development from the spiritual orientation, and emphasized preservice teachers' inherent characteristics and spiritually-oriented career guidance and counseling in teacher education (Liu and Chen, 2020).

### Quality assurance of teacher education

The current research on quality assurance in teacher education documents advanced countries' education systems that emphasize a knowledge framework for teachers, concretise teacher images, construct professional teaching standards, and establish a blueprint for teacher education development (Wu and Tsai, 2017). Taiwan's *Guides for Professional Standards of Teachers* was developed in response to the standards-based trend in quality control for standardized and professional teacher education (Ministry of Education, 2012, 2016; Yen and Pan, 2017). A diversified teacher education system and excellent teacher quality are the main goals of developing standards-based teacher education (Wang, 2017).

### Experimental education and teacher education

In 2014, three experimental education–related acts, namely the Enforcement Act for School-Based Experimental Education, Enforcement Act for Non-School-Based Experimental Education at the Senior High School Level or Below, and Enforcement Act Governing the Commissioning of the Operation of Public Elementary and High Schools to the Private Sector, were passed in Taiwan to implement the provisions of Article 13 of the 2013 Fundamental Educational Act. Several studies have focused on teacher education and teaching quality in experimental education (Ministry of Education, 2013; Chiu and Hu, 2017; Chan, 2019).

### Sociology of teacher education

In recent years, sociological studies have examined teacher education. One such study used the concept of social closure to explore the difficulty and professional credibility of obtaining a teacher's certificate and to establish the professional status, value, and identity associated with a teaching certificate (Huang, 2016b). Another study explored the relationship between Taiwan's Sunflower Student Movement and education through qualitative research, specifically the lenses of critical pedagogy and feminist pedagogy (Yang, 2015). This study also reflected on the educational implications of the student movement.

### Operation of teacher education centers

Only one study focused on the operation of teacher education centers. Chen (2017) studied the webpage marketing of teacher education centers to explore the relationship between the centers' brand images and preservice teachers' loyalty.

In summary, teacher education–related research since the implementation of 12-Year Basic Education has mainly focused on teacher education curricula, international comparisons of teacher education systems, teacher education policies, and internship-related research. New research on teacher education practices, materials and methods, and teacher practicums has been published every year. In addition, relevant research on various educational issues, including test preparation related to teacher education, continues to be published. However, research on the professional development of preservice teachers, teacher education for special education, quality assurance of teacher education, the career development of student teachers, experimental education and teacher education, the sociology of teacher education, and the operation of teacher education centers has remained scarce.

## Suggestions for future research on teacher education

According to our analysis of the issues discussed (and overlooked) in teacher education research after the implementation of 12-Year National Basic Education in Taiwan. The researchers offer the following suggestions for future research on teacher education.

### Research on professional development of preservice teachers

No consensus has been reached on teachers' professional ethics in Taiwan. Professional ethics courses are rarely offered in preservice teacher education programs. According to the standards of professional competence for preservice teachers announced by the Ministry of Education in 2019, teachers' recognition and practice of professional ethics are necessary. However, further research on the alignment of teachers' professional ethics with 12-Year Basic Education is warranted.

### Research on teacher education for special education

Schools have trended toward an integrated education approach for the education of special education students at the senior high school level and below. Teachers play a key role in protecting these students' right to learn. Therefore, teachers' knowledge of special education and of how to cooperate with special education teachers and students' parents are crucial. Although preservice teacher education programs at most teacher education centers offer 3-credit “Introduction to Special Education” courses, few studies have been conducted on improving the quality of teachers' knowledge in special education.

### Research on quality assurance for teacher education

Taiwan's teacher education system is superior to those of many countries in its recruitment and selection process and elements of quality assurance (Ingvarson and Rowley, 2017). The related research has rarely discussed these two themes and their implications. Teacher education research in Taiwan has mainly focused on how to establish and evaluate various standards. The implications and upshots of various standards must be thoroughly discussed. To explore the standards and quality assurance of lifelong learning for teachers and the development of Taiwan's 12-Year Basic Education, social conditions, including declining birth rates, must be considered.

### Research on career development of preservice teachers

A multi-reservation teacher education system was established in 1994. With the establishment of various teacher education universities and declining birth rates, the demand for schoolteachers has decreased, and preservice teachers face challenges in career development and planning. High-quality substitute teachers often have difficulty obtaining teaching jobs, and the quality of work is often questionable. The quality and evaluation of education are also inconsistent among counties and cities, which is unfavorable under the framework of 12-Year Basic Education. The career development of preservice teachers warrants careful attention from researchers and policymakers.

### Research on experimental education and rural schools

Teacher education in experimental education requires more flexible and diversified systems and courses. One of the key goals stipulated in the revised Teacher Education Law in 2019 is to improve the literacy of teachers in experimental education and rural schools. The law aims to provide opportunities for substitute teachers at experimental education schools and rural schools to study preservice teacher education (Ministry of Education, and National Taiwan Normal University, 2019).

The teacher education system for experimental education and education in rural schools has afforded sufficient flexibility. However, research into the content of preservice teacher education courses remains warranted, and determining how to meet the needs and encourage the development of teachers in experimental education and rural schools should remain a priority of researchers.

### Research on sociology of teacher education

Sociological issues related to preservice teachers include teachers' social role, the social nature of teachers' work, the social orientation of teachers' career development, and teachers' social responsibilities. In addition, the effect of various social issues or social events on school teachers and students, as well as the ability of preservice teachers to transform social issues into educational issues and integrate them into learning activities, enhance students' social understanding and sense of participation. In addition, through the study of sociological theory, we can reflect on social factors and influences in the educational context.

### Research on the operation of teacher education centers

Taiwan's teacher education system also adjusted to the new 12-Year Basic Education curriculum by beginning to offer competency-based preservice education courses in 2019. The Ministry of Education required teacher education centers to reconstruct their curricula, and these centers have since developed distinctive courses and increased study activities for preservice teachers to help such teachers develop professional literacy in five key areas. Many preservice teachers who are preparing to select teacher education programs know little about this reform and do not understand what types of professional literacy they must attain through various pre-employment education courses. In the process of operating preservice education courses, teacher education centers must refer to relevant research to understand how to deepen the competency-based curriculum to cultivate the professional literacy of preservice teachers.

## Reflections and conclusion

### Reflections

The national curriculum guidelines of the Republic of China were first established in 1929. Since then, the guidelines for elementary school and secondary school curricula have been revised multiple times to ensure that they are in line with global trends in education. In 1968, 9-Year Compulsory Education was implemented, establishing an excellent foundation for the cultivation of talented individuals in Taiwan. However, identifying methods for alleviating academic pressure to encourage students to further their education and providing holistic education based on the five key dimensions of education (moral, intellectual, physical, group, and aesthetic education) remain crucial concerns in Taiwan. In addition, various major social issues, including a decreasing birth rate, an aging population, diversified interactions between ethnic groups, rapid development of the Internet and information technology, emergence of new job types, increasing democratic participation, growing awareness of social justice, attention to ecologically sustainable development, and transformations brought about by globalization and internationalization, pose numerous challenges to education and contribute to the need for reform to ensure that Taiwan's education system can keep pace with changing social needs and global trends. In addition, Taiwan, a small island in the western Pacific Ocean, has been influenced by international tendencies in terms of its politics, economics, academic research, etc., and the development of teacher education is no exception (Huang, 2010; Ministry of Education, 2014; Shih and Wang, 2022).

Since the implementation of the 12-Year Basic Education in 2014, many major educational reforms and corresponding reforms in teacher education have been enacted. After the implementation of the New Curriculum in 2019, preservice teacher education courses have also moved toward competency-based curriculum reform. Broadly speaking, the teacher education reforms enacted in response to 12-Year Basic Education span four key areas: (1) implementation of the development and design of competency-based preservice education courses; (2) guidance of teacher education universities in the development of school-based teacher education characteristics; (3) encouragement of teacher education universities to ensure the quality of preservice teachers and teaching experience; and (4) development of preservice teachers' inquiry and practical abilities, cross-disciplinary teaching, and international mobility. Therefore, teacher education in Taiwan is closely related to the 12-Year Basic Education curriculum, and the findings of this study may serve as a reference in the effort to promote the quality of teacher education in Taiwan and the sustainable development of Taiwan's education system within the context of the curriculum's implementation.

## Conclusion

This study collected the relevant research on teacher education in Taiwan from 2015 to 2019, evaluated the developments in research on teacher education, and analyzed the themes that have been emphasized in this body of research so far. The main research issues were as follows: (1) preservice teacher education courses; (2) international comparison; (3) teacher education policy; (4) student teachers; (5) teaching materials, methods, and practicums; (6) educational issues; (7) practices in teacher education; (8) test construction; (9) professional development of preservice teachers; (10) teacher education for special education; (11) quality assurance of teacher education; (12) career development of preservice teachers; (13) experimental education and teacher education; (14) sociology of teacher education; and (15) operation of teacher education centers.

Overall, the research on teacher education reflects the focus on curricula and the Taiwanese system of teacher education. However, it still fails to consider all aspects of system reform, such as teacher education for special education and experimental education and quality assurance of teacher education. In addition, studies on the career development and professional development of preservice teachers have remained scarce. Finally, research on teacher education for rural schools, the sociology of teacher education, and the operation of teacher education centers should be prioritized. Regarding our suggestions for future research on teacher education, the body of literature on the following research issues must be further developed: (1) the professional development of preservice teachers, (2) teacher education for special education, (3) quality assurance for teacher education, (4) career development of preservice teachers, (5) teacher education for experimental education and rural schools, (6) sociology of teacher education, and (7) operation of teacher education centers. Our findings may serve as a reference in improving teacher education, and promoting the sustainable development of Taiwan's education system as well as for future research on the theoretical basis and quality of teacher education worldwide.

## Data availability statement

The original contributions presented in the study are included in the article/supplementary material, further inquiries can be directed to the corresponding author/s.

## Author contributions

Conceptualization, methodology, investigation, resources, data curation, writing-original draft preparation, and writing-review and editing: R-JW and Y-HS. Formal analysis: Y-HS. All authors have read and agreed to the published version of the manuscript.

## Funding

This study was funded by the Taiwan Ministry of Science and Technology (Grant No. 110-2420-H-002-003-MY3-Y11014).

## Conflict of interest

The authors declare that the research was conducted in the absence of any commercial or financial relationships that could be construed as a potential conflict of interest.

## Publisher's note

All claims expressed in this article are solely those of the authors and do not necessarily represent those of their affiliated organizations, or those of the publisher, the editors and the reviewers. Any product that may be evaluated in this article, or claim that may be made by its manufacturer, is not guaranteed or endorsed by the publisher.
